# Scolicidal and Apoptotic Activities of 5-hydroxy-1, 4-naphthoquinone as a Potent Agent against *Echinococcus granulosus* Protoscoleces

**DOI:** 10.3390/ph14070623

**Published:** 2021-06-28

**Authors:** Masoud Moghadaszadeh, Mehdi Khayyati, Adel Spotin, Roghayeh Norouzi, Abdol Sattar Pagheh, Sonia M. R. Oliveira, Maria de Lourdes Pereira, Ehsan Ahmadpour

**Affiliations:** 1 Biotechnology Research Center, Tabriz University of Medical Sciences, Tabriz, Iran; masoud.moghadaszadeh@gmail.com; 2 Immunology Research Center, Tabriz University of Medical Sciences, Tabriz, Iran; mehdi.khayyati@yahoo.com; 3 Infectious and Tropical Diseases Research Center, Tabriz University of Medical Sciences, Tabriz, Iran; adelespotin@gmail.com; 4 Department of Parasitology and Mycology, School of Medicine, Tabriz University of Medical Sciences, Tabriz, Iran; 5 Department of Pathobiology, Faculty of Veterinary Medicine, University of Tabriz, Tabriz, Iran; roghayehnorouzi123@gmail.com; 6 Infectious Diseases Research Center, Birjand University of Medical Sciences, Birjand, Iran; satar2011@googlemail.com; 7 CICECO-Aveiro Institute of Materials, University of Aveiro, 3810-193 Aveiro, Portugal; sonia.oliveira@ua.pt; 8 Hunter Medical Research Institute, New Lambton, NSW 2305, Australia; 9 Department of Medical Sciences, University of Aveiro, 3810-193 Aveiro, Portugal

**Keywords:** *Echinococcus granulosus*, scolicidal, nanoliposome, juglone, apoptotic activity

## Abstract

Cystic hydatid disease (CHD) is a zoonotic disease with different clinical stages caused by the larval stage of the cestode *Echinococcus granulosus*. It is important to highlight as a public health problem in various regions of the world. In the current study, the efficacy and apoptotic activity of the liposomal system containing juglone (5-hydroxy-1,4-naphthoquinone) were assessed against protoscoleces (PSCs) in vitro. To this aim, firstly, liposomal vesicles were prepared by the thin-film method. Their physico-chemical features were assessed using Zeta-Sizer and Scanning Electron Microscope (SEM). Subsequently, various concentrations (50, 100, 200, 400, and 800 μg/mL) of juglone nanoliposomes at different exposure times (15, 30, 60, and 120 min) were used against PSCs. Results showed that juglone nanoliposomes at all tested concentrations induced scolicidal effect, however, 800 μg/mL and 400 μg/mL of juglone nanoliposomes could reach 100% mortality in 60 and 120 min, respectively. Additionally, we found that caspase-3 mRNA expression was higher in PSCs treated with juglone nanoliposomes compared to control groups (*p* < 0.001). Therefore, juglone nanoliposomes are suggested to have a more potent apoptotic effect on PSCs. Generally, optimized doses of juglone nanoliposomes could display significant scolicidal effects. Moreover, further in vivo studies are required to evaluate the efficacy of this nanoliposome.

## 1. Introduction

Cystic hydatid disease (CHD) is one of the main neglected helminth diseases, with different clinical complications caused by the larval stage of the cestode *Echinococcus granulosus* in many countries of the world [1]. The metacestode grows as a unilocular cyst that contains an inner germinal layer with totipotent cells that generate capsules with multiple protoscoleces (PSCs) via asexual division, and it is surrounded by a laminated acellular membrane, called the laminar layer [2]. The annual incidence rate of CHD can differ from 1 to 200 per 100,000 populations in numerous endemic areas. The prevalence of CHD in Iran is considered an endemic and hyperendemic area, especially in the southern and northern parts, respectively [3]. CHD has medical and veterinary importance due to broad economic damages and losses of animals [4]. Moreover, the decrease in the quality of meat, milk production, fiber, and the number of surviving offspring are problems of this disease [5]. Humans, sheep, and other mammalian species are intermediate hosts, whereas canids are the definitive hosts for *E. granulosus*. Normally, humans and herbivores get the infection by occasional ingestion of eggs of *E. granulosus* in contaminated food, water, or soil. Oncospheres of eggs are able to penetrate the intestinal mucosa and disseminate through the portal system of the liver and lungs [6]. Vaccination is not a highly effective method for the control of CHD. Although in silico and in vivo studies are being conducted to design vaccines against this parasite [7], to date, there is no appropriate human vaccine against the disease [8,9]. The animal EG95 recombinant vaccine was used for vaccination of sheep against hydatid cyst. Surgery is a routine method for treating the disease, but there are some unexpected side effects, such as anaphylactic shock, disease recurrence, and mortality. Moreover, when cysts are found in the brain and spinal tissues, surgery is not recommended [10,11]. In these cases, chemotherapy and/or puncture-aspiration-injection-respiration (PAIR) technique are alternative resources for the treatment of CHD. Surgery, PAIR technique, and chemotherapy are the most common CHD treatments used today. Removal of the cysts together with chemotherapy, either using albendazole and/or mebendazole before and after surgery, are the best approaches. Nevertheless, some drugs have side effects, such as hepatotoxicity, leucopenia, and thrombocytopenia [12,13].

So far, many natural scolicidal agents have been used to inactivate hydatid cyst PSCs. Among the antiparasite compounds, 5-Hydroxy-1,4-naphthoquinone, also called juglone, is an organic compound with the molecular formula C_10_H_6_O_3_ that is produced both naturally and industrially from different parts of the fruit, bark, leaves, and roots of some species of walnut from *Juglandaceae* family. The scientific name for juglone is *Juglans regia* [14,15]. Nowadays, walnut is widely cultivated across eastern Asia, northern Africa, southern Europe, and western South America. On the other hand, juglone is a phenolic compound with allopathic activity belonging to the class of naphthoquinones. It also has antibacterial, antiviral, anti-fungal, and anti-tumoral activities [16]. Juglone and its derivatives have a broad potent spectrum of antiparasite activity [17,18]. Nanostructured lipid carriers produced containing the drug enhance the penetration of the incorporated compounds and resolve concerns such as side effects, low drug solubility in water, and lack of adequate drug delivery to the parasite [19]. Here, we evaluated the scolicidal and apoptotic activity of nanoliposomed lipid carriers of juglone against *E. granulosus* PSCs in vitro by the qRT-PCR expression of *caspase-3* gene.

## 2. Results

### 2.1. Morphology and Zeta Potential Characterization of Liposomal Systems Containing Juglone

**Figure 1:**
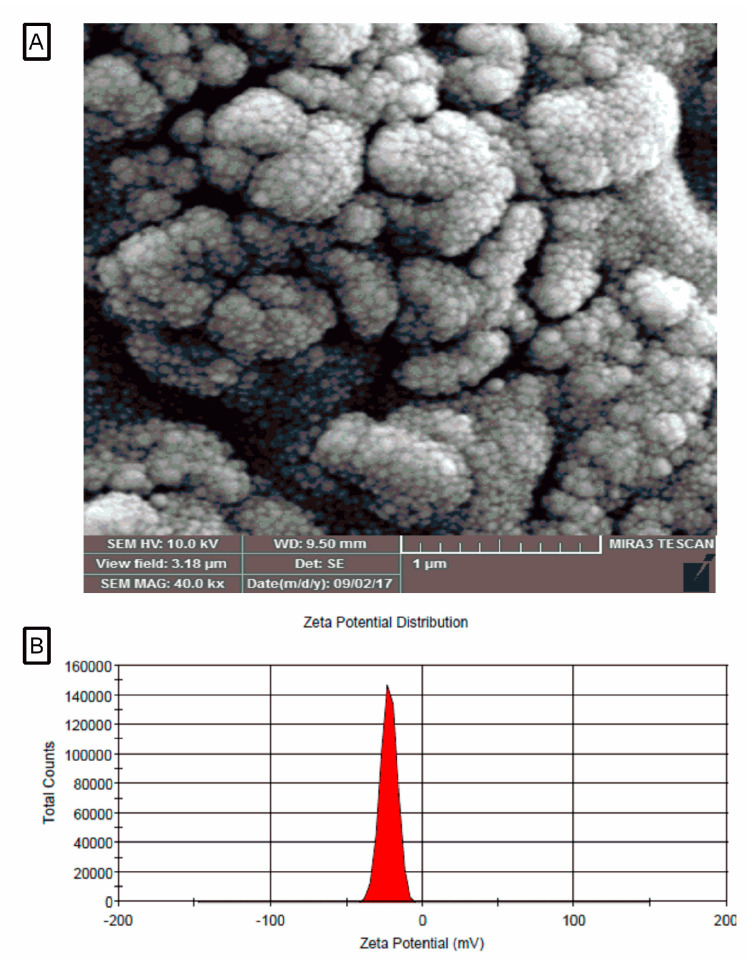
SEM photograph (**A**), zeta potential of the liposomal system containing juglone (**B**).

The morphology of nanoliposome systems containing juglone was investigated by SEM. SEM image shows that the morphology of the constituent particles in liposome systems containing essential oil, are spherical displaying a smooth surface and particles are in the range of 10–90 nm (Figure 1). In addition, the surface charge (zeta potential) of the liposome systems containing the juglone was calculated to be −16.7 mV (Figure 1).

### 2.2. Genotyping of E. granulosus PSCs

To identify the *E. granulosus* PSCs genotype, PCR amplification by targeting the *cox1* gene was performed. Based on sequencing analysis (PouyaGostar Gene, Tehran, Iran) the G1 genotype (sheep strain) was confirmed (data not shown) [20].

### 2.3. Scolicidal Effects of Juglone and Juglone Nanoliposomes

**Figure 2:**
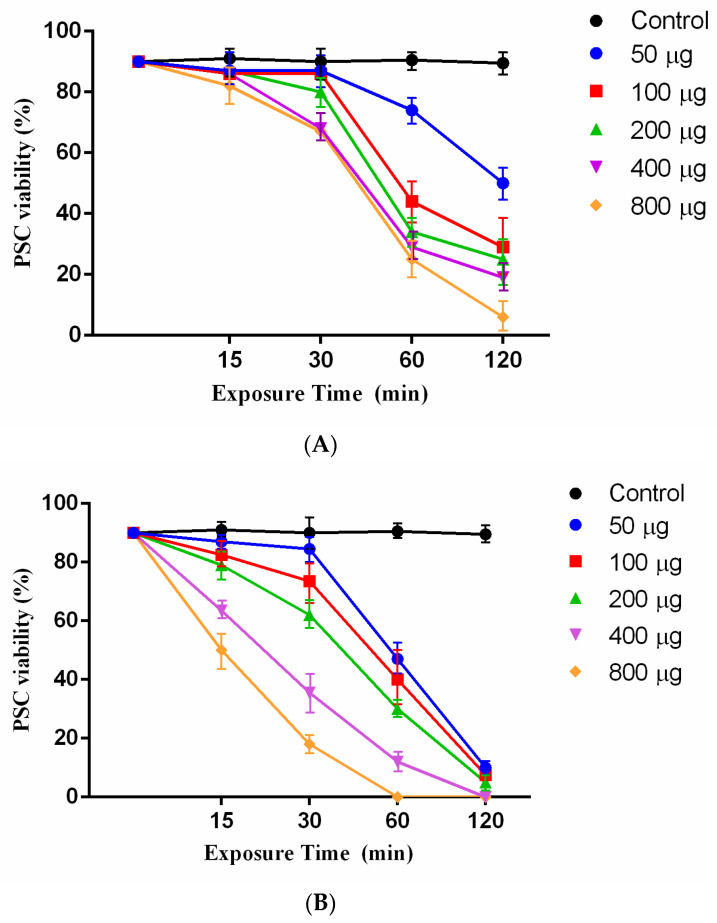
(**A**) Scolicidal effects of different concentrations of juglone at various times of exposure against PSCs of *E. granulosus*. Each test was performed in triplicate. (**B**) Scolicidal effects of different concentrations of the juglone nanoliposomesat various times of exposure against PSCs of *E. granulosus*. Each test was performed in triplicate.

Juglone as an effective agent with various concentrations (50, 100, 200, 400, and 800 μg/mL) was tested at different exposure times (15, 30, 60, and 120 min) against *E. granulosus* PSCs. The results showed that the juglone had a scolicidal effect at all concentrations. Statistically significant differences were observed between 800 μg/mL juglone at exposure times of 120 min (mortality rates of 94%) and the other concentrations and control group (PBS) (Figure 2A). However, the induced scolicidal effect of 50 μg/mL was less than that of other concentrations after 120 min (71%) (Figure 2A).

Remarkably, 800 μg/mL and 400 μg/mL of juglonenanoliposomes could reach 100% mortality at 60 and 120 min, respectively. The scolicidal effect of juglonenanoliposomes at concentrations of 200 μg/mL, 100 μg/mL and 50 μg/mL at exposure times of 120 min were 95%, 92.5%, and 90% mortality rate, respectively (Figure 2B).

### 2.4. Expression of caspase-3 Gene

**Figure 3:**
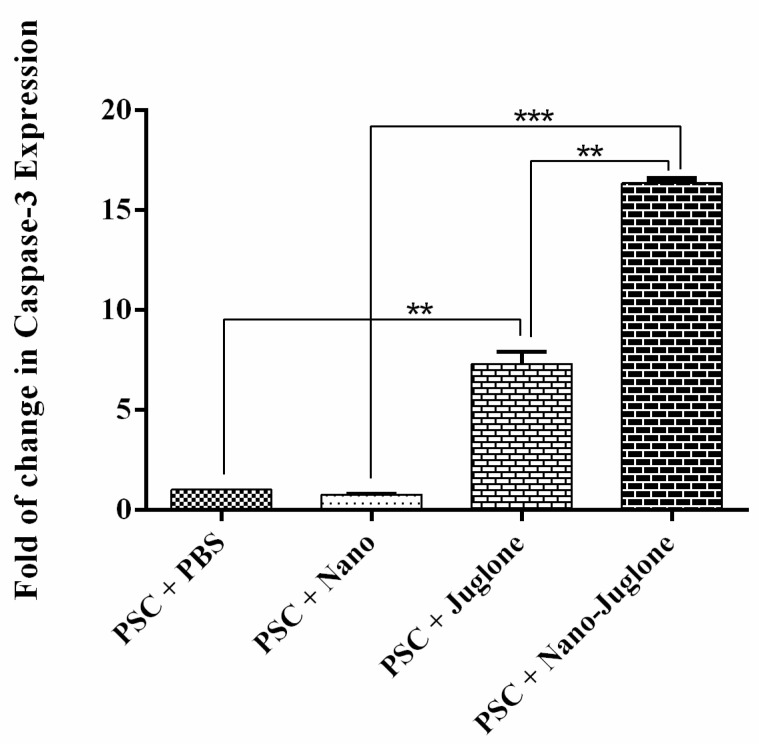
*Caspase-3* gene expression determined by real-time PCR in PSCs treated with PBS (negative control), PSCs treated with nanoliposomes (negative control), PSCs treated with juglone, and PSCs treated with juglone nanoliposomes. The bar graph indicates the mean ± standard deviation. *Caspase-3* mRNA expression was higher in both PSCs treated with juglone nanoliposomes than in control groups (** *p* < 0.01, *** *p* < 0.001).

Apoptotic activity was evaluated using the *caspase-3* mRNA expressions assay. The expression of *caspase-3* mRNA was assessed by the qRT-PCR after 15 h of exposure (Figure 3). As a result, *caspase-3* mRNA expression was higher in PSCs treated with juglone nanoliposomes compared to control groups. However, the rate of apoptosis was significantly different between the PSCs treated with juglone nanoliposomes.

## 3. Discussion

In the present study, the scolicidal and apoptotic activity of juglone and juglone nanoliposomes as a novel agent were successfully established against *E. granulosus* PSCs. The survey showed that 800 μg/mL and 400 μg/mL of juglonenanoliposomes have a more effective scolicidal rate (100% mortality at 60 to 120 min of exposure times), respectively, than the rest of concentrations, while 200 μg/mL, 100 μg/mL, and 50 μg/mL at exposure times of 120 min showed 95%, 92.5%, and 90% mortality rate, respectively. Today, surgery is a nominated method for complicated cases of CHD. However, the success of this method depends on the formation of new cysts, relapse, or secondary dissemination of CHD after surgery, which can cause death due to the leakage of the cyst content [10]. In fact, the inactivation and infertilization of PSCs by scolicidal agents accompanied by minimal side effects and high efficacy instead of opening or removing the cyst are highly recommended [21]. So far, several protoscolicidal agents, such as hypertonic saline, mannitol, chlorhexidine gluconate, huaier aqueous, *Allium sativum*, *Sambucus ebulus*, fungal chitosan, and *Berberis vulgaris* have been used to inactivate the content of hydatid cysts [21,22,23,24]. Unfortunately, the consumption of these agents has been limited because of their low efficacy, toxicity, and undesirable side effects [25].

Hypertonic saline solution (20%) was considered 100% effective in PSCs of hydatid cyst, but acute hypernatremia can cause severe symptoms in the nervous system, such as necrosis, myelinolysis, convulsions, and intracranial bleeding. Silver nitrate and cetrimide have been shown to be 100% effective against PSCs of the hydatid cyst. However, toxic reactions may also be caused by the absorption of these ingredients [26].

It is accepted that apoptosis played a binary role in the association between host and cystic echinococcosis (CE) in the mechanisms of survival and/or suppression [27,28]. Generally, caspase enzymes play a significant role in apoptotic progression. Among them, caspase-3 proteinase is essential for DNA fragmentation and morphological changes associated with cell death. The apoptotic process of praziquantel and dexamethasone was shown in *E. granulosus* PSCs via terminal deoxynucleotidyl transferase dUTP nick end labeling (TUNEL) assay and *caspase-3* enzymatic activity [29,30,31]. Importantly, we found that caspase-3 mRNA expression was higher in PSCs treated with juglone nanoliposomes compared to control groups. Our data are complementary to other observations since it has been found that nano compounds have effective apoptotic activity against *E. granulosus* PSCs. A study has shown that silver nanoparticles as a scolicidal agent can affect *E. granulosus* PSCs [32]. A similar study indicated that sulfoxide-loaded PLGA-PEG and albendazole sulfoxide could act as a novel nanopolymeric particle against *E. granulosus* PSCsvb [32]. There are different causes for the effectiveness of nanoliposomes containing juglone, such as increased penetration of the incorporated compounds, high solubility in water, and adequate drug release. On the other hand, juglone as a natural compound has potential therapeutic effects as well as minor side effects against *E. granulosus* PSCs. 

Indeed, albendazole sulfoxide is the main choice for the treatment of Echinococcosis, however, persuasive evidence indicates that this drug comes with minor side effects, such as alopecia, leukopenia, musculoskeletal pain, pancytopenia, gastric irritation, headache, and elevation in levels of the liver enzymes [33,34,35]. Overall, our findings revealed that the optimized doses of nanoliposomes of juglone can induce significant scolicidal effects. In the future, it would be interesting to discover the apoptotic pathways in CE that affect humans that can assist as targets for the development of new scolicidal drugs. Lastly, the side effects of candidate agents must be studied in cells and also in animal models. 

## 4. Materials and Methods

### 4.1. Preparation of Juglone 

Juglone was purchased from Sigma-Aldrich (CAS Number: 481-39-0), and kept as a 100 mM stock solution in dimethyl sulfoxide at 20 °C for in vitro assays. The solution was centrifuged at 1000 rpm for 5 min. The solution was also filtered through a 0.22 mm millipore syringe filter to remove any impurity before use. Then, different concentrations of juglone (50, 100, 200, 400, and 800 μg/mL) were prepared.

### 4.2. Preparation of Liposomal Systems Containing of Juglone

DL-lactide and glycolide were purchased from Sigma-Aldrich (St. Louis, MO, USA) and recrystallized with ethyl acetate. Stannous octoate (Sn (Oct) 2: stannous 2-ethylhexanoate), nano lipid carriers (molecular weight of 2000, 3000, and 4000), dimethyl sulfoxide, polyethylene glycol (PEGs) and poloxamer 407 were purchased from Sigma-Aldrich. Glyceryl palmitostearate (Precirol^®^ ATO 5) was purchased from Gattefossé (Lyon, France). The nanoliposomes of juglone were prepared using the hot homogenization technique [36]. In this method, the juglone was dissolved in ethanol and added to molten lipidic phase (precirol + myglyol) and mixed completely. Then, the aqueous phase containing the emulsifier was added dropwise to the lipidic phase at the same temperature under homogenization at 20,000 rpm for 20 min. The nanoliposomes of juglone were then produced by solidifying the hot nanoemulsion by cooling to room temperature.

### 4.3. Size and Zeta Potential Characterizationof Juglonein Liposomal Systems

The particle size and polydispersity of the solution were determined using Zetasizer Nano Particle Analyzer (model 3600, Malvern Instruments, Malvern, UK). The nanoliposomes were measured at an angle of 90° and laser light irradiation at 657 nm at 25 °C was used.

### 4.4. Morphology of Liposomal Systems Containing Juglone

The surface morphology of the nanocarriers (roughness, shape, smoothing, and mass) was investigated using a Scanning Electron Microscope (SEM: EM3200, KYKY Technology Development Ltd., Beijing, China).

### 4.5. Collection of E. granulosus PSCs 

Hydatid cysts of *E. granulosus* were obtained from apparently infected sheep livers in an industrial slaughterhouse in East Azerbaijan, northwest of Iran. The hydatid fluid was removed aseptically and transferred to a container and left to set for 30 min. The PSCs were placed at the bottom of the container and then centrifuged at 800 rpm for 5 min. The supernatant was removed, and the yielded PSCs were washed three times with PBS and tested with 0.1% eosin to assess the viability of protoscoleces. Samples of PSCs with viability greater than 90% were selected for further testing. The protoscoleces were left and the live PSCs were stored at 4 °C for further use. 

### 4.6. Genotyping the PSCs

To identify the *E. granulosus* genotype, genomic DNA from the PSCs was extracted using the commercial kit (DNG-plus™ solution; CinnaGen, Tehran, Iran) according to the manufacturer’s instructions. The polymerase chain reaction (PCR) was conducted to amplify the *cox1* (cytochrome *c* oxidase subunit I, accession number: KT154000) in a volume of 25 μL of reaction mixture contained 1 µL of template DNA, 12.5 µL Premix Taq^®^ mix (CinnaGen, Tehran, Iran), l µL of 10 µM of each primer, and 9.5 µL nuclease-free water. Details of the primer sequences used for PCR were described previously [32]. The procedure of PCR amplification consisted of 94 °C for 1 min, 30 cycles of 94 °C for 30 s, 56 °C for 30 s, and 72 °C for 1 min, followed by 72 °C for 10 min, with a final holding step at 4 °C. To identify the PSCs genotype, the amplicons (444 bp) were directly sequenced (PouyaGostar Gene, Tehran, Iran).

### 4.7. Scolicidal Assay

In this study, different concentrations of 5-hydroxy-1,4-naphthoquinone containing 50, 100, 200, 400, and 800 μg/mL were used for different exposure times, including 15, 30, 60, and 120 min. To prepare the mentioned dilutions 50, 100, 200, 400, and 800 μg/mL of agent were dissolved in 1 mL of normal saline in a test tube. Then, the obtained solution was gently mixed. Subsequently, in each experiment, 100 µL of sediment containing 1000 PSCs were added to 100 µL of the solution. After mixing the contents, the test tube was incubated at 37 °C for 15, 30, 60, and 120 min. At the end of incubation periods, in order to assess the viability of PSCs, 10 mL of 0.1% eosin were added to the remaining 20 µL of the PSCs pellet and mixed gently. The stained PSCs were smeared on a manually scaled glass slide, which was covered with a coverslip (24 × 50 mm) and examined under an Olympus BX41TF (Tokyo, Japan) light microscope. Five minutes after the exposure times to the eosin staining, protoscoleces that did not absorb the dye with the movement of the flame cells were verified as potentially viable, otherwise, they were considered as dead PSCs. The percentages of dead PSCs were estimated by counting a minimum of 200 PSCs. The hydatid cyst fluid was considered a negative control group. Besides, 5% NaCl (5 g/100 mL) was used as a positive control group [31]. The experiments were performed in triplicate.

### 4.8. Quantitative Real-Time Polymerase Chain Reaction (qRT-PCR)

The total RNA of the untreated and treated PSCs after 15 h of exposure time was extracted using the RNX Plus Kit (CinnaGen, Tehran, Iran). The amount and purity of the RNA were assessed using NanoDrop 2000c spectrophotometer (Thermo Fisher Scientific, Waltham, MA, USA). Complementary DNA (cDNA) was synthesized using 1 μg of total RNA, random hexamer primer (Thermo Fisher Scientific, USA). In order to evaluate the apoptotic effects of juglone nanoliposomes on PSCs, the specific primers of *E. granulosuscaspase-3* gene were designed by the Oligo Analyzer v.3.1 tool based on reference accession numbers of AB306934 (EF-1α) and LK028577 (*caspase-3*). Primer sequences and cycling conditions were described previously [20,32]. The real-time PCR amplification of the target gene was performed in a 20µL reaction volume containing 10μLof super SYBR green qPCR mastermix (YTA, Iran), 10 pmol of primer and 1μLof cDNA template (0.05–5 ng/µL) by an initial denaturation step at 95 °C for 5 min followed by 35 cycles at 95 °C for 30 s, 58 °C for 40 s, and 72 °C for 45 s (Roche RealTime PCR system, Applied Biosystems). PCR amplification was performed in triplicate to decrease the experimental error. Relative mRNA expression was measured by the 2^−ΔΔCt^ method, and results were evaluated based on thecycle threshold (Ct) value. The beta-actin gene was used as a house keeping gene (internal control) to normalize the expression of the target gene.

### 4.9. Statistical Analysis of Data

All statistical analyses were performed using the GraphPad PRISM software version 6 (GraphPad Software, La Jolla, CA, USA; http://www.graphpad.com, accessed on 11 June 2019). Data for each treatment group were analyzed using the chi-square test. The normality of data was assessed using the Kolmogorov–Smirnov test, and the transformation of data was performed where needed. The one-way and two-way analysis of variance (ANOVA) and Tukey HSD post hoc test were used to assess the statistically significant differences between the means. The *p* values < 0.05 were considered significant. 

## 5. Conclusions

Our results suggest that juglone nanoliposomes have a potent scolicidal effect, and a significant difference in the rate of apoptosis was observed between PSCs treated with juglone and PSCs treated with juglone nanoliposomes. However, further studies are required to evaluate the efficacy of these nanoliposomes in vivo and their clinical applications. 

## Data Availability

Data is contained within the article.
